# There’s an App for That: Content Analysis of Paid Health and Fitness Apps

**DOI:** 10.2196/jmir.1977

**Published:** 2012-05-14

**Authors:** Joshua H West, P. Cougar Hall, Carl L Hanson, Michael D Barnes, Christophe Giraud-Carrier, James Barrett

**Affiliations:** ^1^Computational Health Science Research GroupDepartment of Health ScienceBrigham Young UniversityProvo, UTUnited States; ^2^Computational Health Science Research GroupDepartment of Computer ScienceBrigham Young UniversityProvo, UTUnited States

**Keywords:** mHealth, iPhone, app

## Abstract

**Background:**

The introduction of Apple’s iPhone provided a platform for developers to design third-party apps, which greatly expanded the functionality and utility of mobile devices for public health.

**Objective:**

This study provides an overview of the developers’ written descriptions of health and fitness apps and appraises each app’s potential for influencing behavior change.

**Methods:**

Data for this study came from a content analysis of health and fitness app descriptions available on iTunes during February 2011. The Health Education Curriculum Analysis Tool (HECAT) and the Precede-Proceed Model (PPM) were used as frameworks to guide the coding of 3336 paid apps.

**Results:**

Compared to apps with a cost less than US $0.99, apps exceeding US $0.99 were more likely to be scored as intending to promote health or prevent disease (92.55%, 1925/3336 vs 83.59%, 1411/3336; *P*<.001), to be credible or trustworthy (91.11%, 1895/3336 vs 86.14%, 1454/3349; *P*<.001), and more likely to be used personally or recommended to a health care client (72.93%, 1517/2644 vs 66.77%, 1127/2644; *P*<.001). Apps related to healthy eating, physical activity, and personal health and wellness were more common than apps for substance abuse, mental and emotional health, violence prevention and safety, and sexual and reproductive health. Reinforcing apps were less common than predisposing and enabling apps. Only 1.86% (62/3336) of apps included all 3 factors (ie, predisposing, enabling, and reinforcing).

**Conclusions:**

Development efforts could target public health behaviors for which few apps currently exist. Furthermore, practitioners should be cautious when promoting the use of apps as it appears most provide health-related information (predisposing) or make attempts at enabling behavior, with almost none including all theoretical factors recommended for behavior change.

## Introduction

The use of mobile devices in supporting health behavior change is promising. Aside from expanded opportunities for users to access health information, mobile devices are becoming useful for facilitating the ongoing collection of personal data and cueing behavior change at the most appropriate times [1]. One of the earliest applications was in 1998 when wristwatches were used to cue recovering addicts to complete logs regarding their behavior and feelings [2]. The most recent health applications have focused on a simple message system [3], mostly related to diabetes management [4-6] and smoking cessation [4,7]. Clinical care applications are most prevalent with fewer targeting preventive health behaviors [4].

The recent emergence of smartphones has greatly enlarged both the reach and realm of possibilities for health purposes. In particular, it has provided a platform for developers to design third-party applications (apps), which expand the functionality and utility of these mobile devices. Apps are software programs designed specifically to run on mobile devices. In the “health and fitness” category in Apple’s App Store, developers have created thousands of downloadable apps for Apple’s mobile devices, which include the iPhone, the iPad, and the iPod touch. Since the launch of the Apple App Store in the US in July of 2008, more than 500,000 apps have become available with nearly 25 billion downloads [8]. By 2016, it is estimated that more than 44 billion apps will have been downloaded—which is equivalent to 6 app downloads for every person in the world [9].

**Figure 1:**
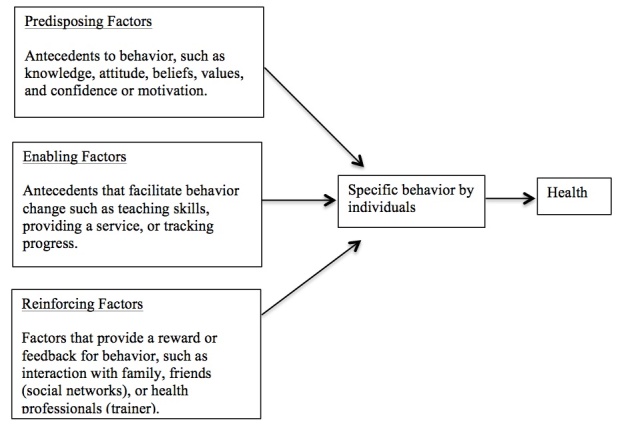
Causal relationship of Precede-Proceed Model factors influencing behavior and health, adapted from the Precede-Proceed Model for health promotion planning and evaluation in Green and Kreuter [12]; apps could address any one of the factors, or in some cases, address multiple factors.

There is growing interest and inquiry in understanding how mobile devices may influence health behavior [10]. Fogg introduced the idea of the functional triad [11]. The triad is a framework that delineates the role of devices in the human–device interaction. According to the triad, devices can be tools, mediums, or social actors. This triad is similar to widely accepted constructs pertaining to the Precede-Proceed Model (PPM) [12], namely predisposing, enabling, and reinforcing factors (Figure 1). Tools, which are similar to predisposing factors, increase the user’s capability. Mediums, which are similar to enabling factors, facilitate an authentic experience for users. Lastly, social actors, akin to reinforcing factors, assist the user in establishing and strengthening relationships. For example, mobile devices become tools or predisposing factors when used to diffuse health information. Similarly, these devices serve as mediums or enabling factors when used by an individual to collect data regarding one’s personal health behavior. Apps can be considered social actors or reinforcing factors because they allow users to interact with social support networks or resources.

Physicians and other health care professionals have begun using many of these apps in their practices, primarily as reference tools [13,14]. As these technologies become more accessible to patients, practitioners may wish to recommend one of the many health and fitness apps to their clients [15,16]. There is currently little analysis in the scholarly literature about the quality of these apps [17]. The purpose of this study was to conduct a content analysis of app developers’ written descriptions of health and fitness apps, which interface with the iPhone. Recognizing that an analysis of these written descriptions falls short of a study design that might entail purchasing, downloading, and using the thousands of health and fitness apps available, the scope of this study aimed to analyze the same information that is available to consumers when considering an app purchase. Many companies develop apps for a variety of mobile devices, including makers of the Android operating system, which now sells more devices than Apple’s iPhone [18]. iPhone apps were chosen for this study because Apple’s App Store still provides the largest selection of apps for download and iPhone users download apps at about twice the rate of Android users, Apple’s closest competitor in the app marketplace [18]. Additionally, this study sought to analyze the written description for each health and fitness app to appraise its potential for influencing behavior change.

## Methods

### Design

This study involved a qualitative content analysis of written descriptions of apps, which were provided by the apps’ developers and were accessed through Apple’s App Store via iTunes. iPhone users can view the App Store using the factory-loaded app, “App Store*.*” The Health Education Curriculum Analysis Tool (HECAT) [19] and the PPM [12] were both used to guide the coding of the apps. The study authors were uniquely qualified to design and conduct this study. The authors had a combined 9 years of experience using iPhone apps for personal use, and 7 years of combined experience with apps in a university setting (eg, conducting research about apps and teaching computer science courses that focus on apps). One of the study authors served on a national planning committee focused on training faculty at institutes of higher education in implementing the HECAT, and three other study authors were trained in health behavior change theory and were very familiar with the PPM. The authors designed the coding methodology and instrumentation following a preliminary review of several hundred health and fitness apps.

The study authors trained two research assistants in the HECAT content areas, the PPM, and study mechanics (eg, data entry and coding the apps). Training sessions were held biweekly over the course of 4 weeks and each session lasted 1 hour. The research assistants were public health undergraduate students in their final year of coursework. Both had completed classes that covered the HECAT content areas and health behavior change theories and models, which included a discussion of the PPM.

### Sample

**Figure 2:**
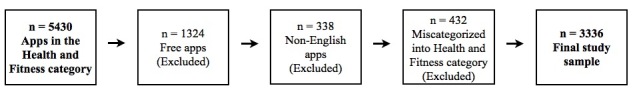
Selection of the study sample.

Apps included in this study sample came from the “health and fitness” category in the US Apple App Store (N = 5430), where descriptions of apps are easily accessed and viewed (Figure 2). The study sample was limited to English language apps within the health and fitness category; therefore, 338 apps were excluded. Freemium apps are a marketing strategy that have an option for free download, offer limited functionality, and then can be upgraded at a cost to the fully functional app. As of September 2011, freemium apps accounted for 72% of app downloads on Apple’s App Store [20]. To avoid the duplication and subsequent coding errors that would arise from coding both the partially and fully functional versions of the same app, this study sample consisted of only paid apps; 1324 freemium apps were excluded. Based upon these inclusion/exclusion criteria, there were 3768 apps available for coding. Of these apps, 432 were miscategorized or misplaced under the health and fitness category (eg, alcohol buyers guides and restaurant locators) and were excluded. The final study sample (n = 3336) was comprised of only those apps that promoted health or prevented disease/injury. Furthermore, 73% of iPhone apps are paid [21]. Paid apps that met the inclusion/exclusion criteria for this study comprised a similar 76% of the total number of apps in the health and fitness category.

### Measurement

Each app was coded for basic descriptive information, such as the app’s name and price. Based upon the preliminary review of apps during study design, the authors concluded that many apps were either not intended to promote health or prevent disease, made claims that were not credible or trustworthy, or could not be recommended to a client for use in a professional setting. Coders assessed each app to determine if, from the perspective of a public health professional, the app was: (1) intended to promote health or prevent disease, (2) credible or trustworthy, and (3) generally recommendable to a client for use to improve health or prevent disease. Examples of apps that were coded as not intended to promote health or prevent disease included such things as restaurant locators or alcohol buyer’s guides. Examples of apps that were coded as not credible or not trustworthy included such things as apps that claimed to predict the gender of your future baby, apps that predict the date of your death, and apps that purport being able to access extrasensory potentials of the mind. Examples of apps that were coded as not being recommendable to clients to improve health or prevent disease included such things as apps for remembering gym locker combinations or tips for applying makeup.

Next, each app was coded and categorized according to the health-related behavior it aimed to influence. Standard health education content areas included in the HECAT were used to facilitate this coding. These content areas were selected because they represent core areas of health education and behavior change, which comprise complete wellness as defined by the Centers for Disease Control and Prevention [19]. The HECAT’s content areas, and subsequent coding categories for this study, included the following: alcohol, tobacco and other drugs, healthy eating, mental and emotional health, personal health and wellness, physical activity, safety, sexual health, and violence prevention. The authors reviewed hundreds of apps during the study design phase in order to inform the creation of the subcategories within each of the core HECAT content areas. After reviewing each app’s description, the coders determined which content areas, and subcategories, were most descriptive of the behavior(s) addressed by the app.

Next, the PPM was used to code each app according to its level of anticipated influence to potentially change behavior. The PPM is a widely accepted health education framework [12] and was used in this study to guide the coding of the apps on several important dimensions related to behavior change. The PPM groups health behavior determinants into 3 main categories: predisposing factors, enabling factors, and reinforcing factors*. *For this study, predisposing apps were those utilities likely to precede behavior and which were cognitive- or affective-based. Predisposing apps were related to: knowledge or awareness of conditions or outcomes (eg, an app that provides cancer statistics); providing information (eg, an app that presents information regarding ways to prevent adverse health outcomes); beliefs, values, or attitudes (eg, an app that discusses common reasons to avoid tobacco in an effort to assist the user in quitting smoking); and confidence or motivation (eg, an app that tries to convince you that you can change your diet). Coders coded apps as enabling if they were intended to be utilized, or occurred, at or around the same time as the desired behavior and if they facilitated behavior through teaching a skill (eg, an app with pictures and instructions on healthy stretching), providing a service (eg, an app that geo-locates places for physical activity), or tracking progress/recording behavior (eg, calorie counter apps). Reinforcing factors are the rewards received and the feedback the learner receives from others following adoption of a behavior, which may encourage or discourage continuation of the behavior. Coders coded apps as reinforcing if they interfaced with a social networking site (eg, apps with automatic upload to Facebook), provided encouragement from trainers/coaches (eg, an app that featured easy communication with a coach or trainer), and included an evaluation based upon the user’s self-monitoring (eg, an app that provided automated feedback about user’s reports of his/her physical activity).

### Analysis

Inter-rater reliability was computed between the 2 coders on a subsample of 10% of the final dataset. The number of agreements were divided by the number of disagreements and the resulting level of concordance was 92%, which is comparable to levels used in previous content analysis research [22]. Disagreements between coders were discussed as a research team until a consensus was reached.

Apps were compared according to price to determine if the basic quality of more expensive apps increased, when compared to apps that cost less than US $1. The price point of US $1 was chosen for comparison because nearly half of the sample (42.3%) of apps was priced below US $1. Frequencies of apps were computed according to each of the HECAT content areas. Additionally, the frequencies of apps were computed according to each of the dimensions of the PPM.

## Results

Most of the apps in this study cost $0.99 (1411/3336, 42.30%), 23.77% cost $1.99 (793/3336), and 33.93% cost $2.99 or more (1229/3336). Table 1 shows the differences between apps according to their listed purchase price. Apps that cost > US $1 ($0.99) were significantly more likely to be coded as intending to promote health or prevent disease (*P *< .001). Higher priced apps were also more likely to be coded as credible or trustworthy at being able to promote health or prevent disease (*P *< .001) and they were more likely to be coded as recommendable to a client for the purpose of improving health or preventing disease (*P *< .001).

Each HECAT content area and the respective representation among study sample apps are included in Table 2. For this study, the HECAT content areas were not mutually exclusive. Indeed, many apps were coded under multiple content areas. Apps related to physical activity and personal wellness were the most common. Among physical activity apps, workout programs and workout monitors accounted for nearly all the apps. Apps in the personal wellness category were comprised of those relating to complementary and alternative medicine, sleep, remedies, disease-specific information (eg, information about heart disease), oral care, and hygiene. Healthy eating and mental health apps combined to account for just over 30% (1065/3336, 31.92%) of apps. Nutritional content of specific food items, calorie tracking or counting apps, and healthy diet apps were the most common under the healthy eating category. In the mental health category, stress management apps comprised the largest subgroup, followed by meditation guides, and remedies, therapies and self-help treatments. Apps related to sexual and reproductive health; alcohol, tobacco, and other drugs (ATOD); and injury prevention were the least common in this study sample. Pregnancy or fertility calendars were the most common type of sexual/reproductive health apps, followed by postnatal care, and then prenatal care. Nearly all ATOD apps were designed to assist the user in his/her own addictions, with 1.53% (2/129) including tips on how to provide support and assistance to an addict. Apps coded under the injury prevention category were mostly related to first aid and emergency preparedness. One-third of injury prevention apps provided information about snakebites.

**Table 1:** Comparison of app credibility by price.

	n (%)			
	$0.99	>$0.99		*P*
This is an app intended to promote health or prevent disease	1411 (83.59)	1925 (92.55)	73.7	< .001
This app is credible or trustworthy	1454 (86.14)	1895 (91.11)	23.3	< .001
As a health care professional, I would use this app for my personal use or recommend it for use by one of my clients	1127 (66.77)	1517 (72.93)	16.9	< .001

**Table 2:** Frequencies of apps according to the Health Education Curriculum Analysis Tool (HECAT) content areas (N = 3336).

HECAT content area		n (%)^a^
**Alcohol, tobacco, and other drugs**		131 (3.93)
	Help for addiction	118 (90.08)
	Support for an addict	2 (1.53)
	Other	17 (12.98)
**Healthy eating**		651 (19.51)
	Calorie counters, journals, logs	213 (32.72)
	Healthy recipes and cooking tips	114 (17.51)
	Healthy diet-specific information	175 (26.88)
	Nutritional breakdown of specific food items	236 (36.25)
	Other	57 (8.76)
**Mental and emotional health**		414 (12.41)
	Eating disorders	11 (2.06)
	Stress management	255 (47.66)
	Depression	41 (7.66)
	Reference tests, diagnostic tools, information	18 (3.36)
	Remedies, therapies, medication guides, self-help treatments	131 (24.49)
	Meditation guides	160 (29.91)
	Other	
**Physical Activity**		1108 (33.21)
	Workouts, tips, ideas	696 (62.82)
	Parks, facilities, directional maps	11 (0.99)
	Race announcements, events	1 (0.09)
	Monitors, measurement of workouts, logs, automatic recordings	496 (44.40)
	Other	8 (0.72)
**Violence prevention and safety**		96 (2.88)
	Attack alarms, notification noises	15 (15.62)
	How-to guide for administering first aid	32 (33.33)
	Information about snake bites	32 (33.33)
	Emergency preparedness	25 (26.04)
	Other	26 (27.08)
**Personal health and wellness**		962 (28.84)
	Sleep	160 (16.63)
	Oral care/hygiene	14 (1.46)
	Disease/illness specific information	146 (15.18)
	Remedies/medications/prescriptions	153 (15.90)
	Goal setting	37 (3.85)
	Beautification	28 (2.91)
	Complementary and alternative medicine	310 (32.22)
	Skin care	31 (3.22)
	Other	156 (16.22)
**Sexual and reproductive health**		243 (7.28)
	Prenatal care	60 (24.69)
	Pregnancy/fertility calendar	82 (33.74)
	Postnatal care	72 (29.63)
	Early parenting strategies and tips	16 (6.58)
	Intimacy enhancer	25 (10.29)
	Other	8 (3.29)

^a ^Apps could be coded in multiple categories.

Most of the apps were coded as either predisposing or enabling (Table 3). Only 6.65% (222/3336) of apps were reinforcing and 1.86% (62/3336) of apps included each PPM factor (ie, predisposing, enabling, and reinforcing). Predisposing aspects of apps mostly involved the provision of knowledge or attempts to raise awareness (713/1776, 40.15%). Enabling apps were most commonly coded as providing a service (1065/2181, 48.85%). Reinforcing apps were coded as such mostly because they interfaced with social networking sites (eg, automatic uploads to Facebook), potentially facilitating feedback from peers regarding behavior. Over 40% (92/222, 41.44%) of reinforcing apps provided some type of feedback—sometimes automated and sometimes human-to-human communication—regarding the user’s self-monitoring of his/her behavior.

**Table 3:** Theoretical classification of apps (N = 3336).

PPM Level		n (%)^a^
**Predisposing**		1776 (53.24)
	Knowledge or awareness	713 (40.15)
	Informative	1372 (77.25)
	Beliefs, values, attitudes	378 (21.28)
	Confidence or motivation	369 (20.78)
**Enabling**		2181 (65.38)
	Teach a skill	810 (37.16)
	Provide a service or sell something	1065 (48.85)
	Track/record behavior	859 (39.40)
**Reinforcing**		222 (6.65)
	Interfacing with social networking sites for encouragement	101 (45.50)
	Encouragement, trainer support, coach	50 (22.52)
	Evaluation based upon self-monitoring	92 (41.44)
**All**		62 (1.86)

^a ^Apps could be coded in multiple categories.

## Discussion

The more expensive apps in this study sample were identified as more credible or trustworthy, more recommendable to clients in a professional setting, and more likely designed to promote health and prevent disease. These findings are consistent with Abroms et al [23] who reported that most of the apps scoring highest in adherence to established clinical practice guidelines for tobacco cessation were paid apps, thus establishing a relationship between quality and price. Together, these findings may indicate that apps with many functions simply cost more. While Heron and Smith [24] reported evidence of mobile devices’ efficacy for bringing about behavior change, the current study supports a recognition that such devices will require a level of sophistication likely to increase cost [25].

Personal health and wellness, physical activity, and (to a lesser extent) healthy eating apps were by far the most represented categories of apps. Each of these categories represent domains in which individuals historically feel empowered to bring about their own change. This may also reflect a perception that solutions to these problems are less complex. Furthermore, the worldwide rate of obesity has more than doubled since 1980 [26], which has perhaps increased the popularity of exercise and nutrition apps that claim to be self-helps for individuals wishing to lose weight. The observation that ATOD apps were less common is of note considering the continued and considerable substance abuse problems in the United States [27-29]. One explanation for this, as Abroms et al [23] point out in their study of smoking cessation apps, is that app users may represent an elite group of affluent individuals, who may be less likely to suffer from substance abuse. As the diffusion of mobile devices increases to include a wider socioeconomic demographic, there may be an increased need for development of efficacious ATOD apps.

More than half of the apps in the current study were established upon predisposing factors, which are primarily knowledge-based. If most health and fitness apps available are simply predisposing factors, professionals should consider the added utility of these apps above and beyond traditional approaches (eg, self-help guides and manuals and reference books). Upon first inspection, apps in the current study appear more affordable (ie, US $0.99) than traditional approaches and are consistent with the price of apps studied in other disciplines [30]. However, considering that all users access the App Store via a device (eg, iPhone, iPod, or laptop) that contracts with a data service provider for a monthly fee, the actual price to the user may indeed be higher, except in cases where users connect using a borrowed connection (eg, work or free Wi-Fi). However, these higher costs may be offset by the convenience associated with being able to consolidate functions into a single device. For example, being able to read about risk factors for heart disease and make personal calls all with the same device may be very appealing to many users. Nevertheless, despite the convenience of modern mobile devices, the health and fitness apps included in this study do not extend beyond what may be accomplished through traditional approaches that employ predisposing factors. Furthermore, given that many of these apps focus on the provision of information, future research should consider the medical accuracy of their content.

**Figure 3:**
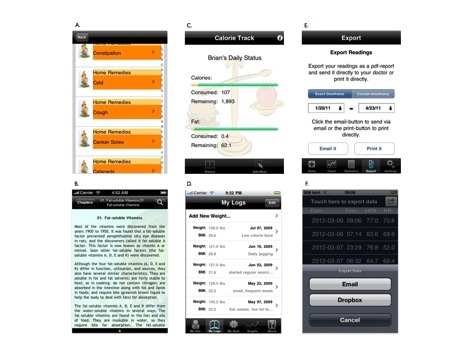
A. iHome Remedies (factor-predisposing); B. The Benefits of Vitamins (factor-predisposing); C. Calorie Track (factor-enabling); D. iWeight Track (factor-enabling); E. DiabetesPlus (factor-reinforcing); F. ithlete (factor-reinforcing).

The most commonly coded theoretical classifications of apps in the current study were those based upon enabling factors, such as teaching skills, tracking progress, or recording actual behavior. This finding is supported by a recent systematic review of mobile device applications for diabetes management, in which Chomutare et al [6] report that tracking progress, an enabling approach, was among the most common features of apps designed for diabetics. Ravert et al [31] note that mobile devices may be used as a means to measure behavior in a convenient and immediate manner, which has historically been dependent on the users recalling and manually inputting data. Indeed, manual uploads to mobile devices regarding one’s behavior may be preferred to more traditional recording practices, namely pen and paper [32]. Additionally, more automated processes introduce the possibility of harnessing the devices’ capabilities for monitoring, which alone may provide incentive to change behavior by allowing the user to monitor and report about their behavior change progress [33,34]. Figure 3 presents actual examples of study apps that represent predisposing, enabling, and reinforcing factors.

Few apps in the current study were found to include reinforcing factors, which are characterized by the provision of encouragement, evaluation, and the opportunity to interact with others. This finding is consistent with that of Abroms et al [23] and Chomutare et al [6] who reported that few apps connect the user to outside sources, including social support systems. This appears to be a missed opportunity given the capacity of emerging mobile device technology. Adapting from the PPM, the hallmark of reinforcing is the extent to which the apps connect the user with external systems or communities, such as social networking sites. As it relates to mobile devices, Patrick et al [1] and Heron and Smith [24] have referred to this process as ecological momentary interventions, or as Intille et al [35] call it, “just-in-time.” Such interventions refer to apps that adapt as a result of data obtained from the user. These uses might be thought of as real-time behavior change support, where users can receive reinforcement via a pre-programmed virtual coach or actual human interaction. According to the PPM, these apps would represent a complete approach to changing behavior and should be most efficacious, which could be determined through research using randomized controlled trials.

Fjeldsoe et al [4] recognized the need for interventions utilizing mobile devices to be based upon theoretical principles. Public health professionals could partner with app developers to create apps that align with established behavior change theories [36], including those that would emphasize the reinforcing paradigm prominent in the PPM. Based upon the current study, the majority of existing apps are limited in their inclusion of reinforcing factors, which are considered vital in facilitating behavior change. Theory is critical in public health interventions and research because it aids in understanding how and why individuals, groups, and organizations behave and change [37]. Public health should take a more active role in the creation of apps, which include theoretical principles. Indeed, previous research has shown that mobile device interventions are most effective when they are based upon theory [3,10]. These interventions will become increasingly more available as the technology’s penetration deepens [38] and could be a powerful tool for the public health professional in broadening their influence and in reaching previously isolated segments of the community [39].

### Limitations

Whereas this study represents one of the first of its kind, there are several limitations that should be considered when interpreting the results. First, the coders used the developers’ own descriptions of their apps in order to code them. It is possible that some developers either overstated or understated the capabilities of their apps, which would have resulted in a misclassification for the purposes of this study. Actually downloading and using the apps was beyond the scope of this study and would not have been feasible for a sample of 3336 apps. Furthermore, the written descriptions of the apps comprise the same information that customers have available to them when they consider making a purchase. Second, the apps represented in this study may not include all of the apps potentially relevant to health and fitness. Just as the coders for this study identified apps that were not intended to promote health or prevent disease, it is possible that other relevant apps were equally misclassified into other App Store categories, which would have precluded their inclusion in this study. In addition, this study focused only on apps associated with Apple’s App Store and excluded apps associated with other platforms (eg, Android apps). Whereas future studies may explore apps designed for other platforms, this study’s focus on iPhone apps was because this platform has the greatest number of apps available for download [40]. Lastly, the PPM and HECAT content areas guided the coding of the apps and appeared adequate to the researchers, but it is unclear to what extent these coding categories covered all of the types of the apps listed under this section in the App Store.

### Conclusions

There are many apps available to those desiring to promote health or prevent disease. Nevertheless, practitioners wishing to recommend the use of third-party apps for such devices as Apple’s iPhone should do so with discretion. It is recommended that practitioners be prudent when promoting the use of apps so as not to overstate their potential effectiveness. Based upon the study apps’ descriptions, it appears that most provide health-related information or make attempts at enabling behavior, with almost none including all factors of the PPM recommended for behavior change. Furthermore, development efforts could target important public health behaviors for which few apps currently exist, such as substance abuse. Future research should extend recent work by Abroms et al [23] and actually test the efficacy of a large number of apps because little is known about their potential health utility.

## Multimedia Appendix 1

Apps coding form.

## Abbreviations

ATOD: alcohol, tobacco, and other drugs
HECAT: Health Education Curriculum Analysis Tool
PPM: Precede-Proceed Model
