# Supplementary material for: There’s an App for That: Content Analysis of Paid Health and Fitness Apps
Source: J Med Internet Res. 2012 May 14;14(3):e72. doi: 10.2196/jmir.1977 (PMC3799565; doi:10.2196/jmir.1977)
Supplement: Supplementary file 1 [file jmir_v14i3e72_app1.pdf]

Multimedia Appendix 1: App coding form

Is this app intended to promote health or prevent disease? (Is this app appropriately categorized in healthcare and fitness (i.e., check no if it is a wine buyer's guide, a restaurant locator, etc.)

1. Yes
2. No

Is this app credible or trustworthy?

3. Yes
4. No

As a health care professional, would I recommend this app for my personal use or for somebody else?

5. Yes
6. No

Alcohol, Tobacco, other Drugs

1. Help for addiction
2. Supporting an addict
3. Shopper's guide
4. Other ____________________

Health eating

5. Calorie counters & food logs/journals/scanners (this is more process related, for example you can enter your own data for the purposes of tracking)
6. Recipes & cooking
7. Diet-specific information
8. Food information (nutrient breakdown of specific food items--information)
9. Other ____________________

Mental and Emotional Health

10. Eating disorder
11. Stress management
12. Depression
13. Reference/Tests/Information (diagnosis tool, information about mental disorders, akin to a DSM, categories or taxonomies of disorders)
14. Remedies/Therapies/Medication (self-help or treatment related options)
15. Meditation

16. Other ____________________

Physical Activity

17. Workouts/Tips/Ideas/Programs
18. Gym locations (parks, facilities, and directional map that provides a location for where to recreate/workout)
19. Race event announcements
20. Monitors/Measurement (workout logs, automatic recordings of how much exercise in terms of distance, intensity, etc. Nikeplus, imap my run)
21. Other ____________________

Safety and Injury Prevention

22. Attack alarms/Notifications (noises that you sound when being attacked, rape violence prevention, alarming your home's alarm system, 911 dialer, EMS locator)
23. First aid (CPR, how to guide for administering first aid, first responder rescue, shock, AED, epipin)
24. Reference/Information (Information about snake bites, bee stings, heat stroke, hypothermia)
25. Emergency preparedness (72-hour kits, food storage guides, emergency contacts and information)
26. Other ____________________

Personal Wellness

27. Sleep
28. Oral care/Hygiene
29. Disease/Illness (infectious/non-infectious, specific information about cancer or diabetes, etc.)
30. Remedies/medications/prescriptions
31. Goal setting (helps you set goals and track them)
32. Beautification
33. Complementary and Alternative Medicine (yoga, pilates, acupuncture, massage, aromatherapy, etc.)
34. Skin care
35. Other ____________________

Sexual and Reproductive Health

36. Prenatal care
37. Pregnancy/fertility calendar
38. STD/Safe sex
39. Post-natal care (Baby health/wellness, Breastfeeding, common baby illnesses, development stages, baby names)
40. Early parenting (parenting strategies, tips, shopping guides, etc.)

41. Intimacy enhancer (Kama sutra, sex positions, sex humor, etc.)
42. Other ____________________

Price

1. .99
2. 1.99
3. 2.99
4. 3.99
5. 4.99
6. 5.99
7. 6.99
8. 7.99
9. 8.99
10. 9.99
11. 10.99
12. 11.99
13. 12.99
14. 13.99
15. 14.99
16. 15.99
17. 16.99
18. 17.99
19. 18.99
20. 19.99
21. Other ____________________

Predisposing-Factors that influence behavior (things that precede behavior and they are cognitive or affective based, like attitudes, knowledge, awareness, beliefs, information, values, confidence, motivation, self-efficacy, etc.)

22. Yes
23. No

Enabling-Factors that influence behavior (Occurs at or around the same time as behavior and it facilitates behavior, Teaching a skill, provide a service, track or record behavior)

24. Yes
25. No

Reinforcing-Factors that influence behavior (Interactive applications, interface with SNS, encouragement from trainers/coaches, you get an evaluation based on your self-report)

26. Yes
27. No

Level of Predisposing

1. Knowledge or Awareness of a health behavior/outcome
2. Informative
3. Beliefs, Values, Attitudes
4. Confidence, Motivation

Level of Enabling

5. Teaches a skill
6. Provides service (sells something, sign-up for events, facilitates engaging in a behavior)
7. Tracking/Recording Behavior

Level of Reinforcement

8. Interfacing with SNS (encouragement from social support)
9. Encouragement-Trainer support, Coach (the app gives you the encouragement, not the network it connects you 2)
10. Evaluation based on self-report
